# Masking Phosphate with Rare-Earth Elements Enables Selective Detection of Arsenate by Dipycolylamine-Zn^II^ Chemosensor

**DOI:** 10.1038/s41598-020-59585-0

**Published:** 2020-02-14

**Authors:** Nutsara Mekjinda, Supho Phunnarungsi, Vithaya Ruangpornvisuti, Raymond J. Ritchie, Itaru Hamachi, Akio Ojida, Jirarut Wongkongkatep

**Affiliations:** 1 Department of Biotechnology, Faculty of Science, Mahidol University, Rama 6 Road, Bangkok, 10400 Thailand; 2 Department of Chemistry, Faculty of Science, Chulalongkorn University, Phayathai Road, Pathumwan, Bangkok, 10330 Thailand; 3 Tropical Plant Biology, Faculty of Technology and Environment, Prince of Songkla University Phuket, Vichitsongkram Road, Kathu, Phuket, 83120 Thailand; 4 Department of Synthetic Chemistry and Biological Chemistry, Faculty of Engineering, Kyoto University, Katsura, Kyoto, 615-8510 Japan; 5 Graduate School of Pharmaceutical Sciences, Kyushu University, 3-1-1 Maidashi, Higashi-ku, Fukuoka, 812-8582 Japan

**Keywords:** Environmental chemistry, Analytical chemistry

## Abstract

Functional reassessment of the phosphate-specific chemosensors revealed their potential as arsenate detectors. A series of dipicolylamine (Dpa)-Zn^II^ chemosensors were screened, among which acridine Dpa-Zn^II^ chemosensor showed the highest capability in sensing arsenate. The presence of excess Zn^II^ improved sensitivity and strengthened the binding between acridine Dpa-Zn^II^ complex to arsenate as well as phosphate. However, due to their response to phosphate, these sensors are not suited for arsenate detection when phosphate is also present. This study demonstrated for the first time that rare-earth elements could effectively mask phosphate, allowing the specific fluorescence detection of arsenate in phosphate-arsenate coexisting systems. In addition, detection of arsenate contamination in the real river water samples and soil samples was performed to prove its practical use. This sensor was further employed for the visualization of arsenate and phosphate uptake in vegetables and flowering plants for the first time, as well as in the evaluation of a potent inhibitor of arsenate/phosphate uptake.

## Introduction

Arsenic is a chemical analog of phosphorus that belongs to the same periodic group and shares a number of similarities with phosphorus, including the same number of valence electrons and nearly identical electronegativity (2.18 for As and 2.19 for P)^1^. Phosphorus- and arsenic-derived oxoanions, importantly inorganic phosphate (Pi) and arsenate, also exhibit similar properties^2^, such as tetrahedral geometry and close bond lengths (1.69A° and 1.52A° for arsenate (HAsO_4_^−^) and phosphate (HPO_4_^−^), respectively)^3^ Their acid counterparts also have similar dissociation constants (p*K*_*a*_ 2.26, 6.76, and 11.29 for the arsenic acid compared with 2.16, 7.21, and 12.32 for phosphoric acid)^1^, thus possessing the same net charge acr.oss pH values. These salient physiochemical similarities to phosphate make arsenate highly toxic to humans. In addition, arsenate is a confirmed carcinogen and the most significant chemical contaminant in drinking-water worldwide^4^.

Detection of arsenate has been conventionally conducted by atomic absorption spectrometry (AAS) and inductively coupled plasma mass spectrometry (ICPMS)^5–10^. However, the techniques require laborious sample preparation and cannot be employed to visualize biological phenomena in *situ*. To overcome this problem, several arsenate-specific chemosensors were developed^11–15^, but they were only compatible with organic or aqueous-organic media and can be unstable in water^16^. Egdal *et al*. (2009) reported the divanadyl complex which was able to selectively bind to arsenate over Pi in aqueous solution, but it was optimal at a slightly acidic pH (pH = 3)^17^. Therefore, for the purpose of arsenate detection in drinking water and biological systems, a chemosensor that is stable in neutral aqueous solution would be more attractive.

Because of the significant roles in biological and environmental systems of the Pi anion, considerable efforts have been devoted to developing methods to detect Pi. These include the well-known colorimetric methods such as molybdenum blue^18–21^ and small molecule-based fluorescence chemosensors, which show promise as analytical tools for the exploration of Pi-related biological processes^22–24^. Compared with the enormous number of reports relating to the detection of Pi, reports of fluorometric arsenate measurement are scant^25^. Owing to their similar chemical properties, we hypothesized that several sensing systems for Pi anion are also applicable to the detection of arsenate anion. We therefore examined the existing chemical sensors developed for Pi detection, as a novel sensing scaffold for arsenate detection. Our developed binuclear zinc complex served as the first turn-on fluorescence molecular sensor for arsenate under neutral aqueous solution^11–15^. However in the arsenate-phosphate coexisting environment, a novel strategy which can suppress the Pi detection while maintaining the arsenate sensing capability would be definitely required. We report a novel strategy of using rare-earth elements to mask Pi and promote specific fluorescence detection of arsenate by small-molecule chemosensors. We found that in the presence of rare-earth elements the binuclear zinc complex exhibited another function as the turn-on fluorescence sensor specific to arsenate with reduced sensitivity toward Pi. The developed sensing system was successfully employed for determination of arsenate contamination in real river water and soil samples contained natural Pi. The aquatic vascular plant *Wolffia* was used as the model plant for the imaging study. The ability to find arsenate contamination in vegetables using a fluorescence microscopy is potentially very important for public health reasons and demonstrated for the first time in this study.

## Result and Discussion

### Arsenate sensing performance

**Figure 1:**
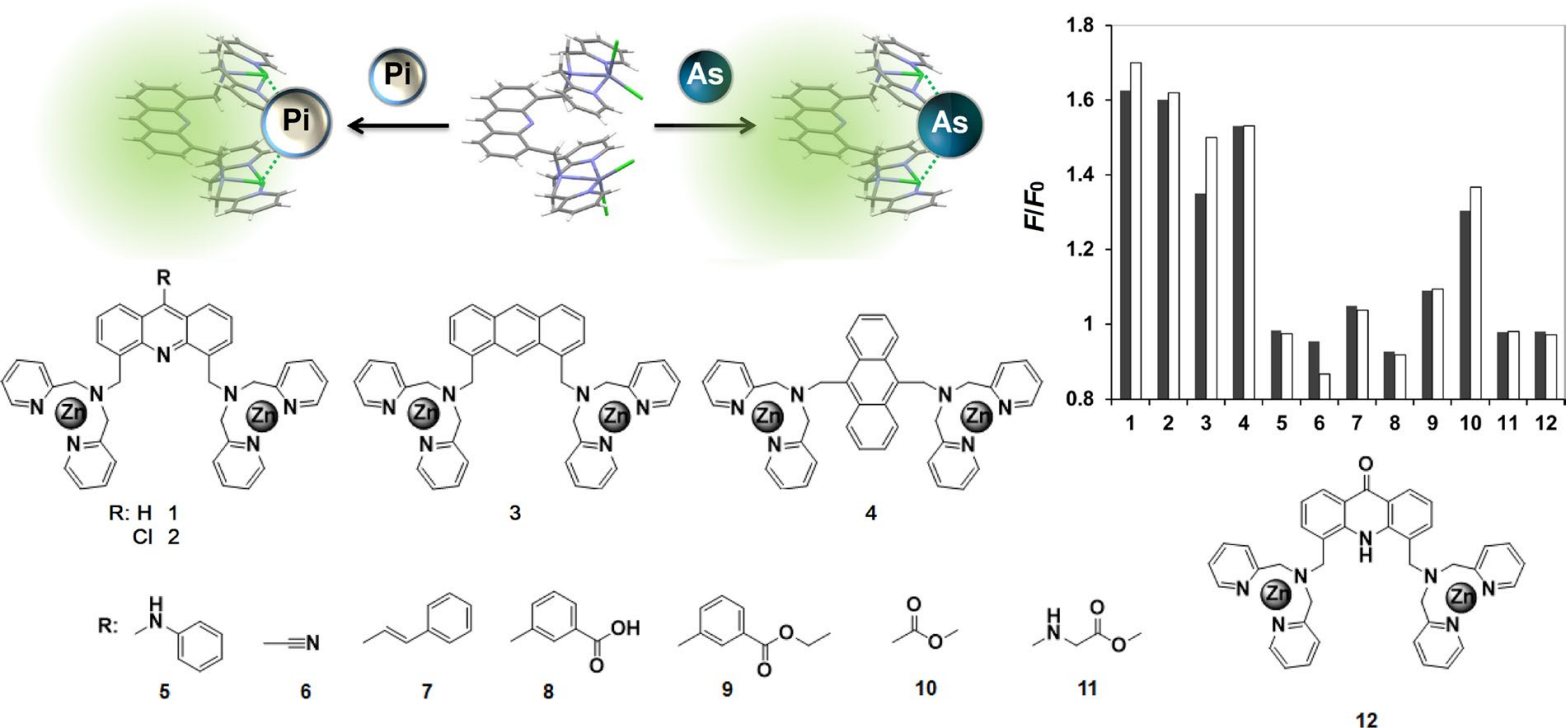
Schematic illustration of the binuclear Dpa-Zn^II^ complex library for fluorescence sensing of arsenate (As) and inorganic phosphate (Pi) and their chemical structures. Bar graph represents the fluorescence change (*F*/*F*_0_) of each compound (5 μM) upon addition of 15 μM of arsenate (gray) or Pi (white).

The binuclear Dpa-Zn^II^ moieties have been studied extensively as an effective binding motif of Pi and its derivatives (Fig. 1)^26^. These complexes have been designed to imitate the binding sites of metalloenzymes, in which the substrate Pi is recognized through reversible coordination to one or more Zn^II^ ions in the binding pocket^27^. Based on density functional theory (DFT) calculations with the B3LYP/6-31 G(d) level of theory, almost similar binding properties of Dpa-Zn^II^ moiety toward arsenate and Pi were found. All the optimized structures of Dpa-Zn^II^ complex after binding to arsenate and Pi, their hydrogen-bond distances, Gibbs free energy of complexations were calculated (see Supplementary Fig. S1, Tables S1 and S2). The results from DFT calculations clearly revealed negligible difference in the binding mode of **1** toward arsenate and Pi. The screening experiment also showed that each of the 12 Dpa-Zn^II^ compounds in our library responded similarly to addition of arsenate and Pi, indicating that these chemosensors were capable of sensing both anionic species (Fig. 1).

**Figure 2:**
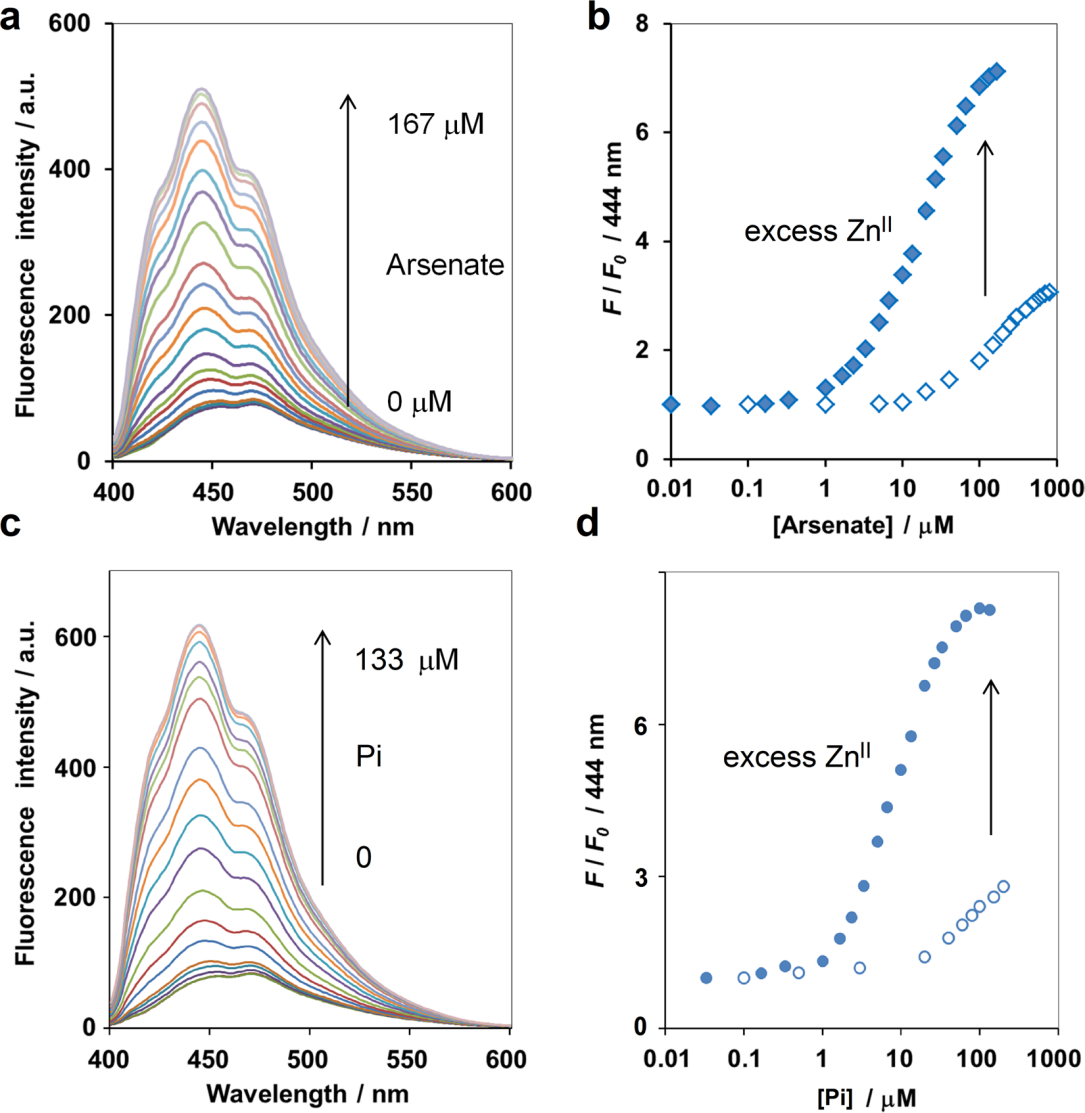
Fluorescence emission of **1** (5 μM) upon addition of arsenate (**a**) and Pi (**c**) in the presence of 0.1 mM ZnSO_4_ and changes in the emission intensity at 444 nm of **1** (5 μM) upon addition of arsenate (**b**) and Pi (**d**) with (filled symbol) and without (empty symbol) addition of 0.1 mM ZnSO_4_. Measurement condition: 10 mM HEPES buffer (pH 7.2), 25 °C. *λ*_ex_ 359 nm.

The compound **1**, in which acridine fluorophore was conjugated to two Dpa-Zn^II^ moieties, showed approximately 1.6-fold increase in fluorescence emission - the highest among the library compounds tested (Fig. 1). It also displayed a hypochromic shift of the maximum emission from 472 nm to 444 nm in 10 mM HEPES buffer (pH 7.2) upon addition of arsenate and Pi (Fig. 2). These changes were similar to the result that Yoon *et al*. (2007) reported this sensor as a sensing motif for Pi^26^. When Zn^II^ was supplied in excess, the fluorescence intensification of the **1** upon arsenate addition was significantly increased from 3-fold to 7-fold as shown in Fig. 2b and summarized in Table 1. Similar phenomenon was observed in the case of Pi addition (Fig. 2d).

**Table 1:** Summary of the Apparent Binding Constant (*K*_app_, M^−1^) and the Maximum Relative Emission Intensity (*F*_max_/*F*_0_) of the Selected Sensors under Excess Zn^II^ Condition Tested in this Study.

Sensor	*λ*_ex_/*λ*_em_ (nm)	Arsenate		Pi	
		*K*_app_ (M^−1^)	*F*_max_/*F*_0_	*K*_app_ (M^−1^)	*F*_max_/*F*_0_
1	359/444	2.7 × 10^5^	7.1	1.4 × 10^6^	8.3
2	368/451	8.3 × 10^5^	3.4	4.2 × 10^5^	4.4
3	370/421	2.2 × 10^5^	1.6	5.5 × 10^5^	1.6
4	380/435	1.2 × 10^4^	2.0	3.6 × 10^4^	2.6

In aqueous HEPES buffer (pH 7.2), the observed emission maximum (see Supplementary Fig. S2) and the shape of the UV/Vis spectrum (see Supplementary Fig. S3) indicated that **1** existed predominantly as a mononuclear Zn^II^ complex with coordination between the Zn^II^ ion and the acridine nitrogen atom. The complexation constants of the first and second Zn^II^ ions of **1** in aqueous HEPES buffer were determined by the Zn^II^ titration experiments to be >10^7^ and 3.3 × 10^4^ M^−1^, respectively, suggesting that the second Zn^II^ ion would not be fully complexed at low micromolar concentrations, which was in good agreement to the case of **2** reported previously^22^. The 20-equivalent excess of Zn^II^ to **1** supplied in this study could offset the low value of the complexation constant of the second zinc ion and enable the formation of binuclear **1** complex capable of arsenate sensing. Addition of excess Zn^II^ also suppressed the fluorescence background of **1** by 44%. As a result, the fluorescence increase of **1** upon arsenate addition was as high as 7-fold in the presence of excess Zn^II^ compared with only 3-fold when Zn^II^ was not in excess (Fig. 2b). Thus, the presence of excess Zn^II^ could drastically increase the sensitivity of **1** for both arsenate and Pi detection, as well as decrease the lower detection limit of **1** toward arsenate from 10 to 4 μM.

The increase in fluorescence emission and the blue shift of **1** due to arsenate were almost identical to those obtained with Pi addition (Fig. 2), indicating that both arsenate and Pi may interact with **1** through a similar mechanism. The Job plot between **1** and arsenate revealed 1:1 stoichiometry (see Supplementary Fig. S4), similar to that of **1** and Pi, and in good agreement with the previous report^26^. From the fluorescence titration under excess Zn^II^ condition, the apparent binding constant (*K*_app_) between **1** and arsenate was 2.7 × 10^5^ M^−1^ as determined by the least-square curve-fitting method (Table 1), while the corresponding value for Pi binding was calculated to be 1.4 × 10^6^ M^−1^. Yoon *et al*. (2007) reported the *K*_app_ between **1** and Pi to be 9.34 × 10^4^ M^−1^ −15-fold lower than the *K*_app_ calculated under the excess Zn^II^ condition used in this study^26^. The observed difference in *K*_app_ suggested that the presence of excess Zn^II^ not only enhanced the sensitivity of the fluorescence detection, but also strengthened the binding affinity between **1** and the target anion. Possibly, this may be because coordination of the second Zn^II^ is required to poise the conformation of the binuclear **1** complex for arsenate and Pi sensing.^1^H NMR spectra suggested the possibility of the coordination between acridine nitrogen atom and the first Zn^II^ but such coordination was cancelled when the second Zn^II^ was fully coordinated with the Dpa moieties (see Supplementary Fig. S5).

The selectivity test confirmed the high sensitivity of **1** toward arsenate and Pi, but not toward arsenite and other anions commonly found in nature such as chloride, bromide, iodide, sulfate, acetate, and inorganic pyrophosphate (see Supplementary Fig. S6). Such selectivity was in good agreement with the previous reports of Hamachi *et al*.^22^ and Yoon *et al*.^26^. However, while this selectivity against other ions was desirable for arsenate detection in real environmental samples, the inability of **1** to distinguish between Pi and arsenate would still be problematic due to the abundance of the former in nature. Therefore, a novel strategy to preclude Pi binding is essential to improve the accuracy and precision of arsenate detection under the Pi-abundant conditions.

### Masking with rare-earth element

**Figure 3:**
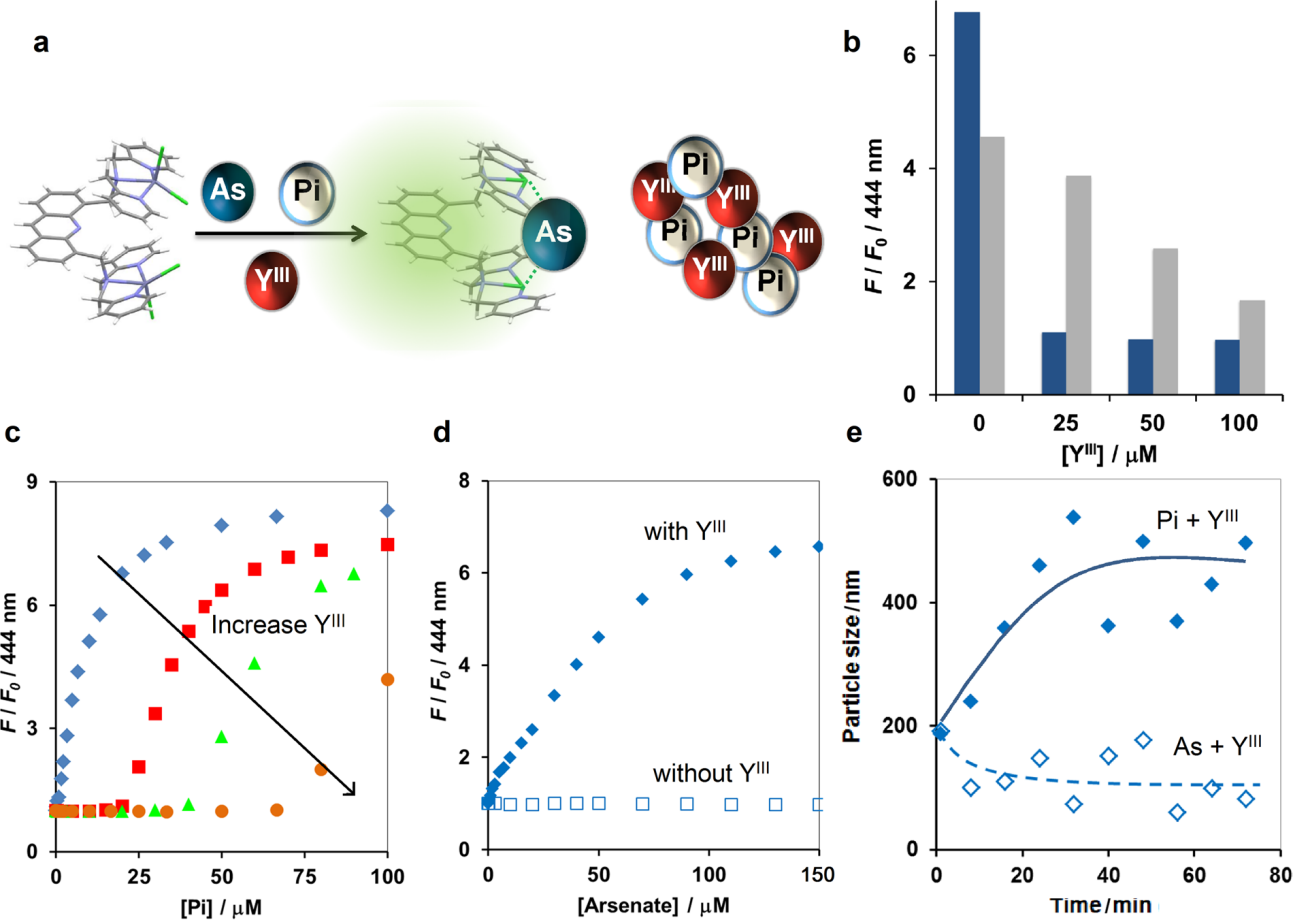
(**a**) Schematic illustration of masking with Y^III^ for detection of arsenate (As) in the presence of Pi. (**b**) Fluorescence change at 444 nm of **1** (5 μM) upon addition of 20 μM arsenate (gray) or Pi (blue) at different concentration of Y^III^. (**c**) Fluorescence change at 444 nm of **1** (5 μM) upon addition of different concentration of Pi when Y^III^ was added at the concentrations of 0 (blue diamond), 25 (red square), 50 (green triangle) and 100 μM (brown circle). (**d**) Fluorescence sensing of arsenate using **1** (5 μM) in the presence (filled diamond) and absence (empty square) of 50 μM Y^III^ as a masking agent when Pi (25 μM) was present. Measurement condition: 10 mM HEPES buffer (pH 7.2), 0.1 mM ZnSO_4_, 25 °C. *λ*_ex_ 359 nm. (**e**) Time-course DLS analysis of the nanoaggregates after equimolar of arsenate (empty symbol) or Pi (filled symbol) was added to the aqueous solution of Y^III^.

As rare-earth elements were reported to show stronger affinity toward Pi than arsenate, we sought to test their efficacy as novel masking agents of Pi in 100% aqueous solution and at neutral pH. Yttrium (Y^III^) exhibits an approximately 100-fold difference in *K*_sp_ between YPO_4_ and YAsO_4_ (p*K*_sp_ are 24.76 and 22.60, respectively)^28,29^. Therefore, Y^III^ was employed as a new type of masking agent of Pi for detection of arsenate using **1**. The rationale is that when both arsenate and Pi are present, Y^III^ will selectively form aggregate with Pi while arsenate will remain soluble and can be detected by the sensor (Fig. 3a).

In the absence of Y^III^, chemosensor **1** was more selective towards Pi than arsenate. However, when 25 μM of Y^III^ was included, **1** became more specific to arsenate than Pi. The higher concentration of Y^III^ suppressed the *F*/*F*_0_ of **1** toward Pi at the baseline while it slightly lowered the fluorescence response of **1** to arsenate (Fig. 3b). At fixed 25 μM of Y^III^, addition of Pi up to nearly 25 μM did not raise the fluorescence level of **1** from the baseline. This result indicated that Y^III^ and Pi formed a nanostructure with 1:1 stoichiometry, leaving free Pi at a concentration below the detection limit of **1** (i.e. below 1 μM Pi). Addition of Pi at concentration higher than 25 μM induced an increase in *F*/*F*_0_ of **1**, suggesting that the free Y^III^ ion had been depleted due to formation of YPO_4_ and could no longer sequester the oncoming Pi. Increasing in the concentration of Y^III^ allowed more Pi to be effectively masked (Fig. 3c), indicating that this masking effect strongly depended on the concentration of Y^III^. However, at high concentration of Y^III^, the masking capacity of Y^III^ slightly dropped, as 100 μM of Y^III^ could mask Pi only up to approximately 75 μM (Fig. 3c). This deviation requires further investigation.

When arsenate and Pi were simultaneously present, Y^III^ did not appear to mask arsenate, as indicated by approximately 6-fold fluorescence enhancement upon addition of arsenate to the aqueous solution containing **1** and 25 μM Pi. In the absence of Y^III^, 25 μM of Pi (5 equivalents to **1**) was able to saturate **1** and resulted in the lack of fluorescence sensing capability upon addition of arsenate (Fig. 3d). Dynamic light scattering (DLS) also confirmed that mixing Y^III^ with Pi led to the formation and growth of YPO_4_ nanostructures, which reached the final size of 451 ± 87 nm, while no growth of YAsO_4_ was observed under a similar condition (Fig. 3e). Taken together, these results demonstrated for the first time that the formation of YPO_4_ nanoaggregate is an effective Pi masking strategy in the arsenate-Pi coexisting system.

**Figure 4:**
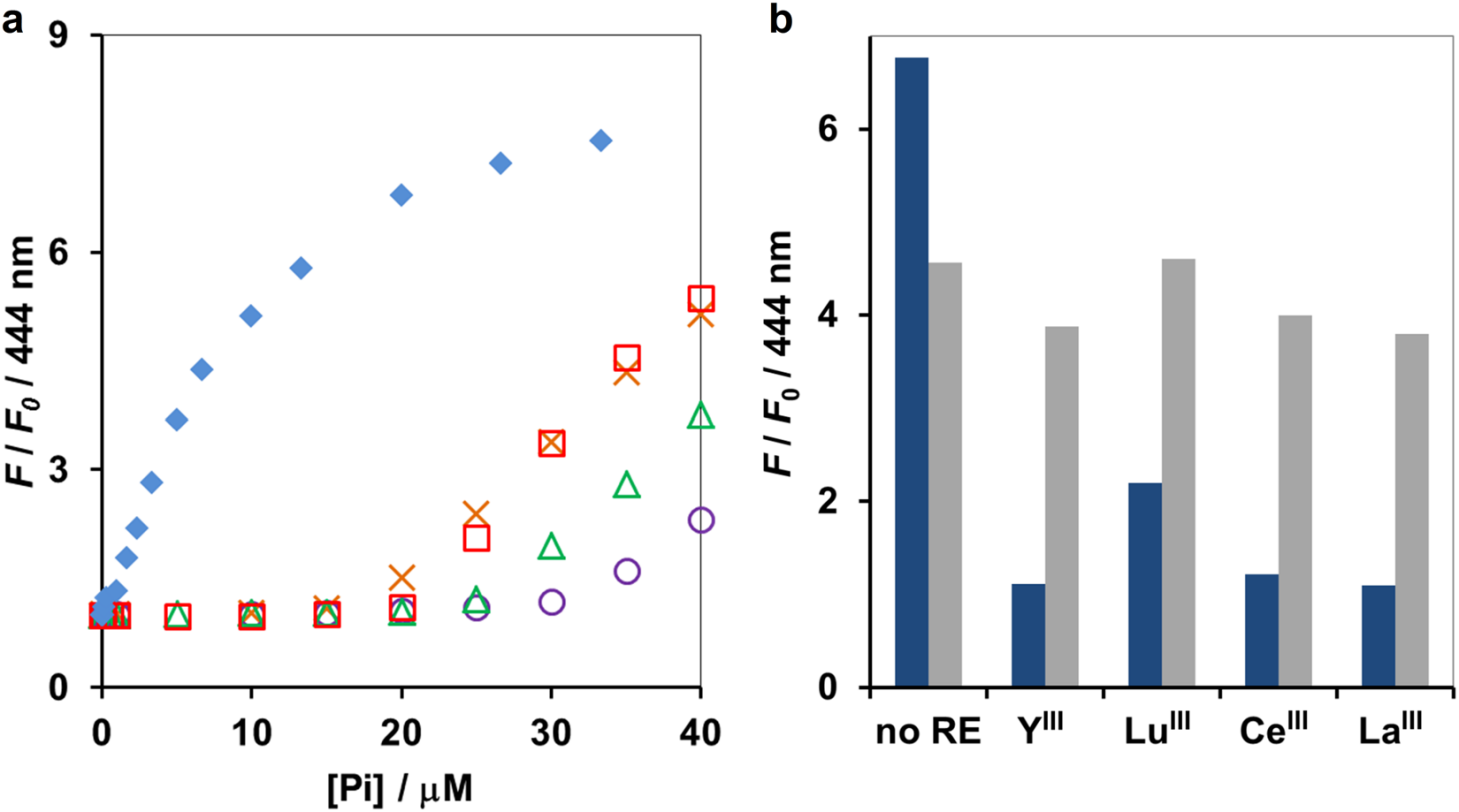
(**a**) Fluorescence change at 444 nm of **1** (5 μM) upon binding to Pi without (blue diamond) and with 25 μM of Y^III^ (red square), Lu^III^ (orange cross), Ce^III^ (green triangle), La^III^ (purple circle) as a masking agent. (**b**) The comparison of *F*/*F*_0_ at 444 nm of **1** (5 μM) upon binding to 25 μM arsenate (gray) and Pi (blue) under 25 μM of each rare-earth element. Measurement condition: 10 mM HEPES buffer (pH 7.2), 0.1 mM ZnSO_4_, 25 °C. *λ*_ex_ 359 nm.

In addition to Yttrium, other rare-earth elements including Lanthanum (La), Cerium (Ce) and Lutetium (Lu) exhibited a similar masking effect when tested under the same condition (Fig. 4a). The fluorescence response of **1** toward arsenate was unaffected by addition of Y^III^, La^III^, Ce^III^ and Lu^III^, suggesting that rare-earth elements did not disturb the sensor’s arsenate detection capability (Fig. 4b). The values of *K*_app_ between **1** and arsenate in the presence of rare-earth elements ranged from 3.2 × 10^4^ M^−1^ in the case of Lu^III^ to 5.1 × 10^4^ M^−1^ in the case of Ce^III^ (Table 2) and was not significantly different among species of rare-earth elements tested. In addition, rare-earth elements could still effectively mask Pi when the chemosensor was changed to **2** and **3**, suggesting that this strategy was relatively independent of the types of probe. However, under high concentration of Y^III^ (150 μM), arsenate binding was weakened and the *K*_app_ between **1** and arsenate diminished to 3.0 × 10^3^ M^−1^, an order of magnitude less than the *K*_app_ at low concentration of Y^III^ (25 μM) (Table 2). Collectively, these results suggested that rare-earth elements were generally effective in masking Pi without compromising arsenate-sensing ability, although at excessive concentrations they could be detrimental to arsenate binding. While high Pi content can be found in the environmental samples, which may necessitate correspondingly high concentration of the masking agent, natural Pi contents in water resources and soil solutions rarely exceed 4 and 10 μM^30,31^. Therefore, 25 μM Y^III^ should be sufficient for effective masking of phosphate in analysis of normal environmental samples.

**Table 2:** Summary of the Apparent Binding Constant (*K*_app_, M^−1^) of Selected Sensors toward Arsenate in the Presence of Rare-earth Elements.

Sensor	*K*_*app*_ (M^−1^)				
	Y^III^		Ce^III^	La^III^	Lu^III^
1^a^	[Y^III^]				
	25 μM	3.8 × 10^4^	5.1 × 10^4^	4.4 × 10^4^	3.2 × 10^4^
	50 μM	1.4 × 10^4^	—	—	—
	100 μM	8.3 × 10^3^			
	150 μM	3.0 × 10^3^			
2^b^	1.9 × 10^4^		2.5 × 10^4^	3.1 × 10^4^	1.5 × 10^4^
3^c^	1.8 × 10^4^		3.2 × 10^4^	2.1 × 10^4^	1.5 × 10^4^

^a^Measurement condition: 5 μM **1**, 0.1 mM ZnSO_4_, 10 mM HEPES (pH 7.2), 25 °C, *λ*_ex_ 359 nm. ^b^Measurement condition: 50 μM **2**, 0.2 mM ZnSO_4_, 200 μM rare-earth element, 10 mM HEPES (pH 7.2), 25 °C, *λ*_ex_ 368 nm. ^c^Measurement condition: 5 μM **3**, 50 μM rare-earth element, 10 mM HEPES (pH 7.2), 25 °C, *λ*_ex_ 370 nm.

### Detection of arsenate in the river water and soil samples

**Figure 5:**
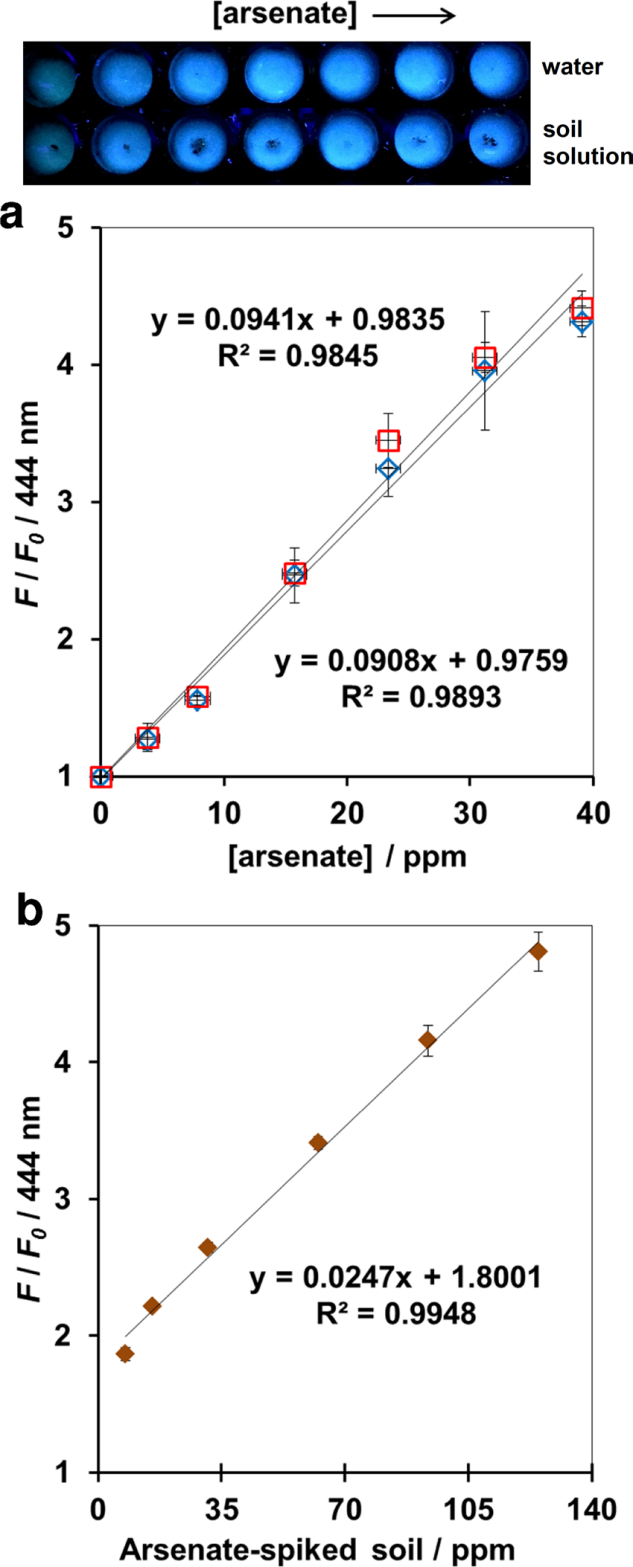
Fluorescence change at 444 nm of **1** (5 μM) when the following samples were added: (**a**) Na_2_HAsO_4_ spiked river water 1 (red square) or river water 2 (blue diamond); (**b**) water-extractable phase of soil spiked with Na_2_HAsO_4_. Measurement condition: 10 mM HEPES buffer (pH 7.2), 25 μM of Y(CH_3_COO)_3_, 0.1 mM ZnSO_4_, 25 °C. *λ*_ex_ 359 nm. Three individual replicates were performed.

Tens millions of people in south and southeast Asia are considered to be at risk from consuming water that has unsafe arsenic levels. It has been suggested that arsenic is naturally released from near-surface, river-derived sediments and transported over centuries through the underlying aquifer back to the river^32,33^. Arsenate is a common form of arsenic usually found in water supplies^34^. Using **1** for detection of arsenate in environmental samples such as river water and soil was successfully demonstrated as shown in Fig. 5. This method also showed good linearity (R^2^ > 0.989) when **1** was applied to the artificially spiked river water as well as the water-extractable portion of soil in a 96-well plate format. These results confirmed the utility of **1** in high-throughput arsenate detection in a complex environmental sample containing natural Pi, which is a major interference for an accurate measurement of arsenate concentration in spectrophotometric analysis.

### Bioimaging of arsenate and Pi in *Wolffia*

**Figure 6:**
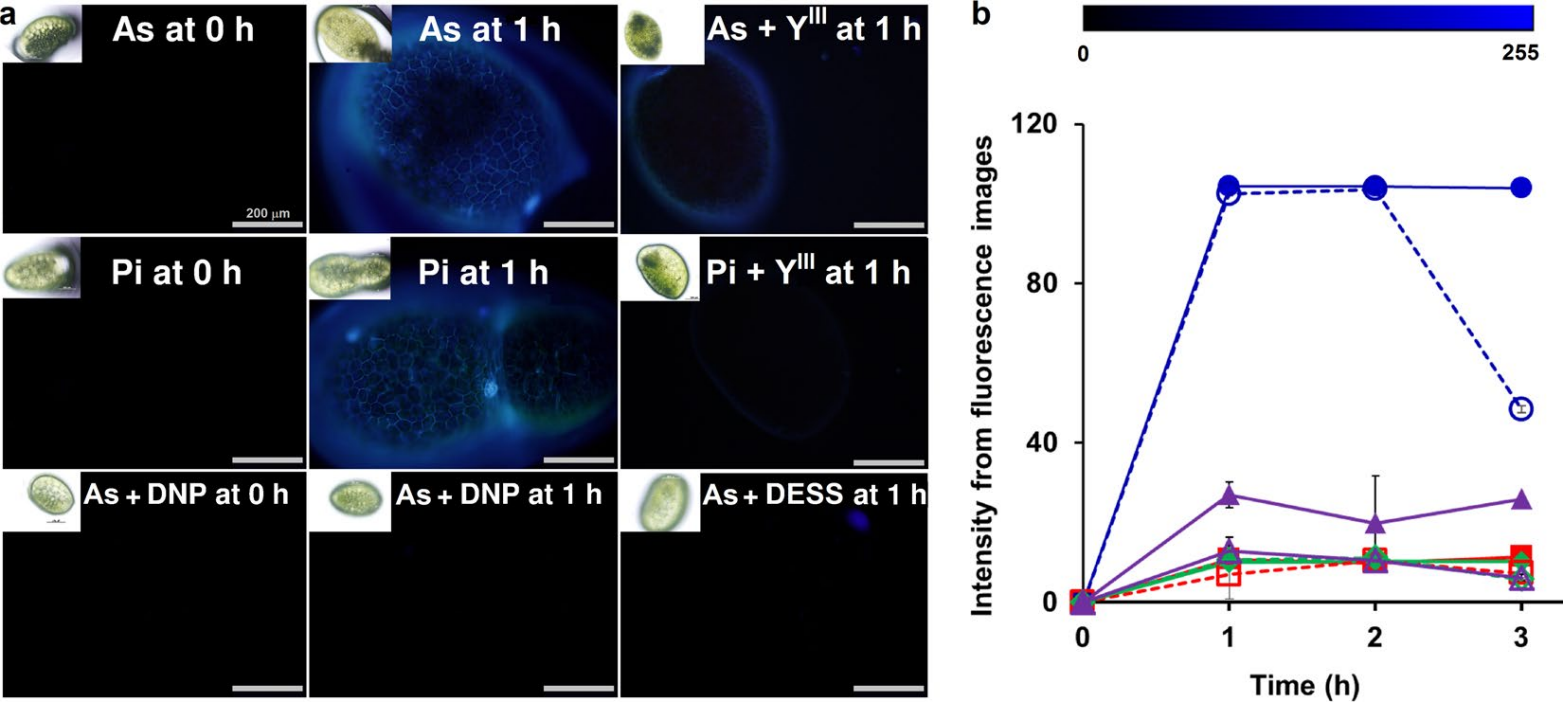
(**a**) Fluorescence images of *Wolffia arrhiza* when incubated with 1 mM Na_2_HAsO_4_ (As), 1 mM Na_2_HPO_4_ (Pi), and 0.1 mM DNP, 0.1 mM DESS or 0.05 mM Y^III^. All specimens were subsequently incubated with 1 mM of **1**. Measurement condition: 20 mM ZnSO_4_, 27 °C, exposure time 1/5.0. Scale bar: 200 μm. (**b**) Average intensities of fluorescence imaging of *Wolffia arrhiza* stained with **1** after treatment with 1 mM arsenate (filled symbols) and 1 mM Pi (empty symbols) in the absence (blue circle) and presence of 0.1 mM DNP (red square), 0.1 mM DESS (green diamond) or 0.05 mM Y^III^ (purple triangle). Data acquired from three individual replicates.

Arsenate accumulation in rice^9,35–38^, fruit and flowering plants^39–41^ is considered a major problem for consumers. It has been suggested that uptake of arsenate in plants relies on PHT phosphate transporters, a specific family of plant plasma membrane proteins^42–44^, but the detailed mechanism has not yet been elucidated due to the lack of an appropriate visualization tool. The improved sensitivity of **1** upon excess Zn^II^ condition encouraged us to apply this small-molecular probe for the bioimaging of arsenate and Pi in plant for the first time. *Wolffia* (*Wolffia arrhiza*), the flowering plant species consumed as a vegetable in South East Asia was selected as a model study. The bright blue color of **1**/arsenate and **1**/Pi complex were detected only when arsenate or Pi was introduced to the *Wolffia* as clearly shown in Fig. 6a. Bright fluorescence from **1**/arsenate and **1**/Pi colocalized with the signal from toluidine blue O staining, suggesting that the complex may be located at the carboxylated polysaccharide accumulated in the cell wall^45^ (see Supplementary Fig. S7). The images further revealed that the depletion of Pi in *Wolffia* occurred faster than arsenate, as the bright fluorescence from **1**/Pi complex disappeared at 3 h post Pi addition, whereas the signal from **1**/arsenate remained for more than 3 h (Fig. 6b). Image analysis revealed that the signal arising from the arsenate-sensor complex exhibited the maximum intensity of 104.02 ± 2.02 at one hour after incubation, then remained almost unchanged throughout 3 h of observation. However, the fluorescence emission from **1**/Pi achieved the maximum intensity of 103.61 ± 1.07 after two hours before decreasing to 48.44 ± 0.96 at the 3^rd^ hour (Fig. 6b). Addition of Y^III^ exhibited the higher masking efficiency toward Pi rather than As (Fig. 6), indicating that rare-earth element can be employed for masking of Pi under imaging analysis. Inhibition of arsenate uptake could also be visualized with **1**, as inclusion of photonophore, 2,4-dinitrophenol (DNP) and diethylstilbestorol (DESS), a reported inhibitor of PHT1 phosphate transporter^42^, significantly decreased the observed fluorescence intensity (Fig. 6a, lower panel). This finding strongly supported the hypothesis that arsenate acquisition depends on PHT1 phosphate transporter^42^. Taken together, these results confirmed the utility of **1** in bioimaging and screening of inhibitors of both arsenate and Pi uptake in vegetable and flowering plants under physiological condition. Previously, conventional methods such as radioactive ^31^P labeling or genetically encoded fluorescent protein have been employed to study arsenate and Pi uptake through PHT phosphate transporter family in live plants^46,47^. However, to the best of our knowledge, our work is the first to use a small-molecule sensor to study this physiological arsenate uptake process *in situ*. Our proposed method can be applied in high-throughput screening of arsenate uptake inhibitors, which will be useful to prevent arsenic contamination in the edible plants so that they are safer for human consumption.

## Conclusion

In this study, we confirmed another function of some small molecular-based chemosensors consisting of Dpa-Zn^II^, which have been reported as a Pi sensor, as a new sensing platform for arsenate and its applicability for visualization of both arsenate and Pi uptake in plants, and for inhibitor screening of the PHT1 transporter in vascular plants. High-throughput screening for a potent inhibitor which prevents arsenate uptake but does not disrupt Pi acquisition in plants could be performed easily using our newly proposed sensing platform to save consumers from ingestion of arsenic-polluted vegetables and fruits. We also demonstrated that the rare-earth masking strategy is a versatile method to suppress the interference of Dpa-Zn^II^ complex-based chemosensor by Pi and allow for specific arsenate detection, enables the sensing of arsenate in natural Pi-arsenate coexisting systems such as river water and soil samples. Because the arsenic contamination in environment is a spatial and temporal problem, a routine monitoring of arsenate level in water supplies in high-throughput manner would be another cost-effective strategy. While our study focused on Dpa-Zn^II^ sensors, we believe that this strategy is applicable to a broad range of highly sensitive chemical and bio-based sensors. Currently, for example, we are investigating the use of this masking strategy to convert a phosphate binding protein into an arsenate specific protein sensor, the outcome of which will be reported soon.

## Methods

### Chemicals and materials

All chemicals used in this work were of analytical reagent grade and used without further purification. Water used in all experiments was doubly distilled and purified by a Milli-Q system. Yttrium(III) acetate (99.9%, (CH_3_COO)_3_Y·4H_2_O), cerium(III) acetate (Ce(CH_3_COO)_3_·H_2_O), lanthanum(III) carbonate (La_2_(CO_3_)_3_), lutetium acetate (99.9%, (CH_3_COO)_3_Lu·4H_2_O) and zinc acetate (99.9%, (CH_3_COO)_2_Zn·2H_2_O) were obtained from Wako (Osaka, Japan). Disodium hydrogen arsenate (Na_2_HAsO_4_·7H_2_O) and 4-(2-hydroxyethyl)-1-piperazineethanesulfonic acid (99.5%, HEPES) were purchased from Sigma-Aldrich (St. Loius, USA). Disodium hydrogen phosphate (Na_2_HPO_4_·12H_2_O) was acquired from Ajax Finechem (Taren Point, Australia). 2,4-dinitrophenol (DNP) and diethylstilbestorol (DESS) was purchased from Tokyo Chemical Industy (Tokyo, Japan). The synthesis of acridine skeleton chemical sensors **1**, **2** and anthracene skeleton chemical sensors **3**, **4** was performed following the literature^22,26,48^. Compounds **5–12** were obtained from Yoshifumi Miyahara’s compound library of Hamachi Laboratory, Kyoto University.

### Fluorescence measurement

A spectrofluorometer (Jasco FP 6500, Japan) was used for the determination of fluorescence intensity of the chemosensors upon binding with arsenate/phosphate. The excitation wavelength was determined based on the fluorometric property of each chemosensor. An automated microplate reader (TECAN, Spark 10 M, Switzerland) was used for the analysis in the 96-well plate format, particularly in the application of arsenate detection in soil and water samples. Fluorescence imaging was performed using fluorescence light microscope (Olympus BX51) with a camera set (Olympus PM10-SP) and UV filter. All spectra were recorded at 25 °C. Intensity of the acquired images was evaluated using Olympus cellSens Software dimension desktop 1.18.

### Dynamic light scattering measurement

Y(CH_3_COO)_3_ was mixed with equimolar Na_2_HPO_4_ or Na_2_HAsO_4_ in a MilliQ purified water (resistivity <18 MΩ, conductivity 0.055 mS/cm) and filtered with 0.2 μm nylon membrane. The particle size of the nanoaggregate was recorded by Zetasizer Nano ZS (Marlvern Instrument, UK).

### Preparation of soil and water samples

Water and soil samples used in this study were spiked with an appropriate concentration of disodium hydrogen arsenate (Na_2_HAsO_4_). Water artificially contaminated with arsenate was prepared by mixing the stock solution of disodium hydrogen arsenate (1.5 mM) with river water 1 and 2 which were collected in Bangkok, Thailand on 10 March 2018. Preparation of arsenic-contaminated soil sample (1.0 g) was performed by spiking the sandy loam soil (surface soil collected from Mahidol University, Phayathai campus, Bangkok Thailand) with arsenate and incubated at 25 °C for 3 days. Soil samples were then suspended in a MilliQ deionized water (10 mL) for 1 h and centrifuged (12,000 × g at 15 min) to obtain a clear supernatant as a water-extractable fraction of soil.

### Preparation of *Wolffia* sample

*Wolffia* (*Wolffia arrhiza*) was commercially available from a local market in Bangkok, Thailand and grown in exterior tubs in commercial NPK medium with the pH adjusted to 5. Then it was soaked in 1 mM of disodium hydrogen arsenate or 1 mM disodium hydrogen phosphate without and with 0.1 mM DNP and DESS for 0, 1, 2 and 3 h at 27 °C. Then, the sample was further incubated in the solution of chemosensor **1** (1 mM) containing ZnSO_4_ (20 mM) for 30 min in dark at 27 °C. The incubated *Wolffia* was transferred to a concave glass slide for fluorescence imaging.

## Supplementary information

Supplementary Information.
