# Supplementary material for: Masking Phosphate with Rare-Earth Elements Enables Selective Detection of Arsenate by Dipycolylamine-ZnII Chemosensor
Source: Sci Rep. 2020 Feb 14;10:2656. doi: 10.1038/s41598-020-59585-0 (PMC7021768; doi:10.1038/s41598-020-59585-0)
Supplement: Supplementary file 1 — Supplementary Information. [file 41598_2020_59585_MOESM1_ESM.pdf]

Supplementary Information

Masking Phosphate with Rare-Earth Elements Enables Selective Detection of Arsenate by Dipycolylamine-Zn^{II} Chemosensor

Nutsara Mekjinda[1], Supho Phunnarungsi[2], Vithaya Ruangpornvisuti[2], Raymond J. Ritchie[3], Itaru Hamachi[4], Akio Ojida[5,*], and Jirarut Wongkongkatep[1,*]

[1]Department of Biotechnology, Faculty of Science, Mahidol University, Rama 6 Road, Bangkok 10400, Thailand

[2]Department of Chemistry, Faculty of Science, Chulalongkorn University, Phayathai Road, Pathumwan, Bangkok 10330, Thailand

[3]Tropical Plant Biology, Faculty of Technology and Environment, Prince of Songkla University Phuket, Vichitsongkram Road, Kathu, Phuket 83120, Thailand

[4]Department of Synthetic Chemistry and Biological Chemistry, Faculty of Engineering, Kyoto University, Katsura, Kyoto 615-8510, Japan

[5]Graduate School of Pharmaceutical Sciences, Kyushu University, 3-1-1 Maidashi, Higashi-ku, Fukuoka 812-8582, Japan

List of Figures and Tables

Figure S1. The orbital plots of HOMO-1, HOMO, LUMO and LUMO+1 for **1** and its complexes with arsenate and Pi

Figure S2. Fluorescence emission of ligand of **1** titrated with Zn^{II}

Figure S3. UV/Visible spectrum of ligand of **1** titrated with Zn^{II}

Figure S4. Job's plot between **1** and arsenate/phosphate

Figure S5. ^{1}H NMR (DMSO-d6) of the acridine Dpa ligand (30 mM) titrated with Zn^{II}

Figure S6. Staining of *Wolffia* with toluidine blue O in comparison with **1**

Figure S7. Fluorescence emission of **1** upon addition of arsenate, Pi and other potential interferences

Table S1. Energies and thermodynamic properties of the complexations of **1** with arsenate and Pi

Table S2. Frontier orbitals HOMO, LUMO, energy gap, electronic-transition wavelengths (λ), oscillator strengths (f), and corresponding transition contributions of **1** and their complexes in water

DFT calculation

To gain more insight into the molecular natures of **1** (denoted Dpa/Zn_2), complexes of **1** with Pi (HPO_4^{2-}) and arsenate ($HAsO_4^{2-}$) were investigated using the density functional theory (DFT) calculations. The hybrid density functionals, Becke's three-parameter exchange functional combined with the Lee-Yang-Parr correlation functional (B3LYP) using the 6-31G(d) basis set and the solvent-effect of polarizable continuum model (PCM) using the CPCM (conductor-like PCM, water as a solvent) model with UFF molecular cavity model, called the CPCM/B3LYP/6-31G(d) method were employed in the calculations. The CPCM/B3LYP/6-31G(d)-optimized structures of **1**, complexes with Pi and arsenate ions in aqueous solution were obtained. All reaction energies and thermodynamic properties of complexations were derived from the frequency calculations of all related species at the same level of theory. The electronic excitation of the Dpa/Zn complexes were obtained from TD-DFT method at the CPCM/B3LYP/6-31G(d) level of theory. All calculations were performed with the Gaussian 09 program.[1]

The CPCM/B3LYP/6-31G(d)-optimized structures of **1** and complexes with Pi and arsenate in aqueous solution were obtained. The CPCM/B3LYP/6-31G(d)-optimized structure of **1** was found to be in good agreement with the X-ray crystallographic geometry at 120 K.[2] The orbitals HOMO-1, HOMO, LUMO and LUMO+1 for the sensor **1** and its complexes with Pi and arsenate ions are shown in Fig. S3. The complexation energies, enthalpy and Gibbs free energy changes of **1** with Pi and arsenate ions, are shown in Table S1. Based on the Gibbs free energy changes of complexations, chemosensor **1** complexing with Pi and arsenate ions are in preference order: HPO_4^{2-} > $HAsO_4^{2-}$.

According to the CPCM/B3LYP/6–31G(d)-optimized structures of **1** complexes with Pi and arsenate ions, **1** was found to bind with Pi or arsenate ion using its zinc atoms. The double binding modes of **1** toward HPO_4^{2-} and $HAsO_4^{2-}$ were found with the hydrogen-bond distances of 2.22 Å and 2.53 Å for HPO_4^{2-} and of 2.21 Å and 2.38 Å for $HAsO_4^{2-}$. These short bond distances indicate the formation of strong intermolecular hydrogen bonds.

The electronic excitations of **1** and their complexes with HPO_4^{2-} and $HAsO_4^{2-}$ are shown in Table S2, respectively. The Table S2 shows that the electronic transition wavelengths are slightly overestimated by 15-20 nm, compared with the experiment.[3] The major transition contributions of **1** and its complexes are the wavelengths in the range 384-388 nm which are the electronic transitions corresponding to HOMO to LUMO states as shown in Fig. S3.

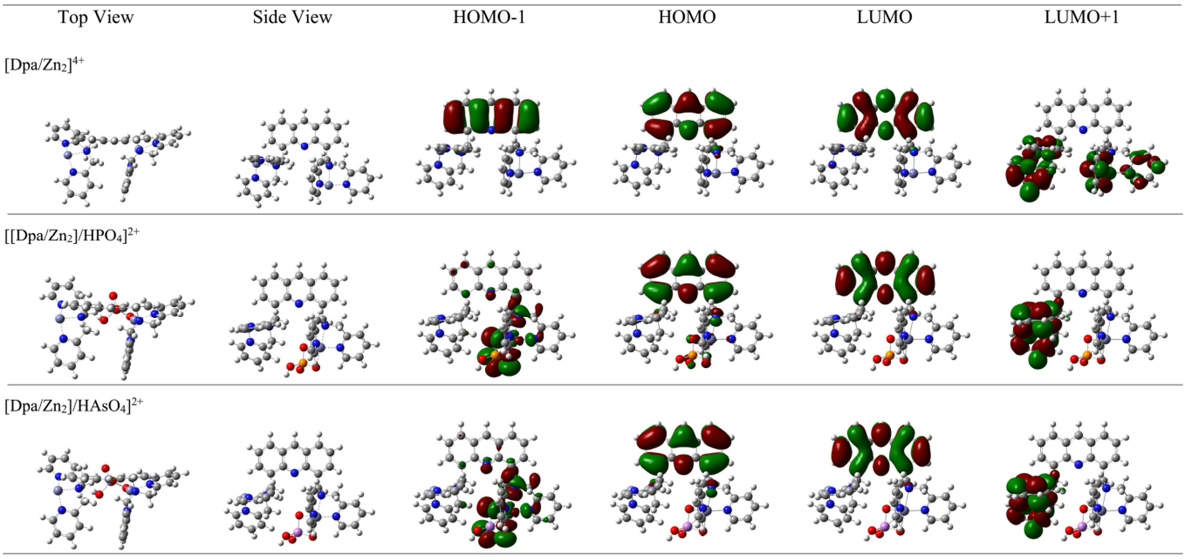

Figure S1. The orbital plots of HOMO-1, HOMO, LUMO and LUMO+1 for **1** (denoted Dpa/Zn_2) and its complexes with Pi and arsenate ion.

Complexation	ΔE_{ZPE} [a,b]	ΔH_{298} [b]	ΔG_{298} [b]
$[Dpa/Zn_2]^{4+} + HPO_4^{2-} \rightarrow [[Dpa/Zn_2]/HPO_4]^{2+}$	-72.83	-72.29	-60.24
$[Dpa/Zn_2]^{4+} + HAsO_4^{2-} \rightarrow [[Dpa/Zn_2]/HAsO_4]^{2+}$	-89.32	-88.72	-76.68

[a] Zero-point energy correction.

[b] Energies in kcal/mol.

Table S1. Energies and thermodynamic properties of the complexations of **1** (denoted Dpa/Zn_2) with Pi and arsenate ions, computed at the CPCM/B3LYP/6-31G(d) level of theory.

Species	HOMO [a]	LUMO [a]	E_{gap} [b]	λ [c]	f [d]	Major transition contributions [e,f]
$[Dpa/Zn_2]^{4+}$	-0.22761	-0.09451	3.622	384.69	0.1098	H→L (98%)
				318.77	0.0822	H-1→L (68%), H→L+11 (17%)
				244.63	0.5457	H-3→L+4 (29%), H→L+11 (23%), H→L+12 (22%)
$[[Dpa/Zn_2]/HPO_4]^{2+}$	-0.22246	-0.08978	3.610	386.41	0.1123	H→L (98%)
				369.34	0.0047	H-1→L (96%)
				318.13	0.0725	H-5→L (11%), H-4→L (55%), H→L+9 (17%)
				247.70	0.5637	H-7→L+1 (12%), H-6→L+2 (16%), H→L+9 (10%), H→L+11 (17%)
$[[Dpa/Zn_2]/HAsO_4]^{2+}$	-0.22225	-0.0895	3.612	386.01	0.1166	H→L (98%)
				354.81	0.0023	H-5→L (10%), H-1→L (85%)
				318.02	0.0729	H-5→L (11%), H-4→L (56%), H→L+9 (21%)
				247.89	0.6533	H-6→L+2 (12%), H→L+9 (21%), H→L+11 (12%)

[a] In au. [b] In eV. [c] In nm. [d] Oscillator strength of a transition in dimensionless.
[e] The contributions, ≥10% are shown. [f] H and L represent HOMO and LUMO, respectively.

Table S2. Frontier orbitals HOMO, LUMO, energy gap, electronic-transition wavelengths (λ), oscillator strengths (f), and corresponding transition contributions for sensor **1** (denoted Dpa/Zn_2) and their complexes in water. The results were computed at the CPCM/B3LYP/6-31G(d) level of theory.

References

1. Frisch, M. J., Trucks, G. W., Schlegel, H. B., Scuseria, G. E., Robb, M. A., Cheeseman, J. R., et al. Gaussian 09; revision A. 1; Gaussian, Inc.: Wallingford CT, 2009.
2. CCDC-605914 contains the supplementary crystallographic data for this paper. These data can be obtained free of charge from The Cambridge Crystallographic Data Centre at www.ccdc.cam.ac.uk/structures
3. Ojida, A. *et al.* Design of dual-emission chemosensors for ratiometric detection of ATP derivatives. *Chem. - An Asian J.* **1**, 555–563 (2006).

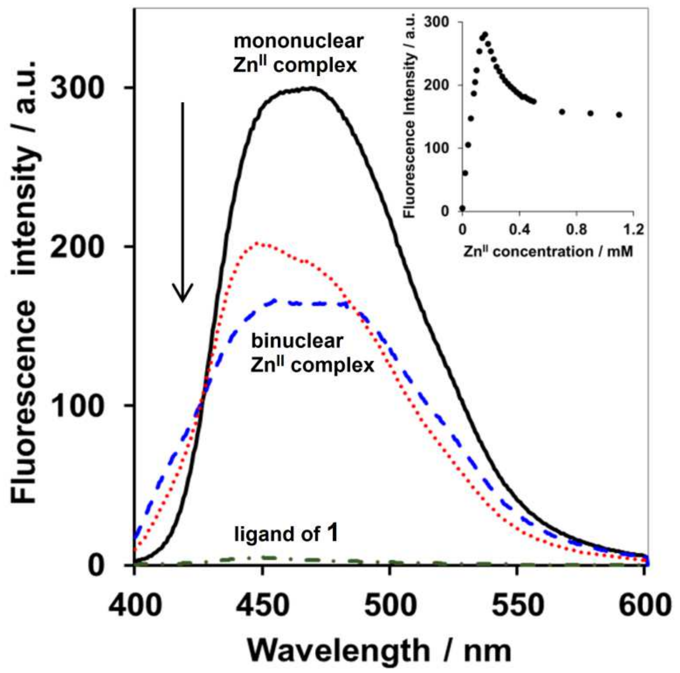

Figure S2. Fluorescence emission of ligand of **1** (0.16 mM) titrated with Zn^{II} at concentration of 0 (green dash dot line), 0.16 (black solid line), 0.32 (red round dot line) and 3.2 mM (blue dash line). Plot of fluorescence intensity at 444 nm (inset) upon Zn^{II} titration in 10 mM HEPES buffer/MeOH = 1/1 (v/v).

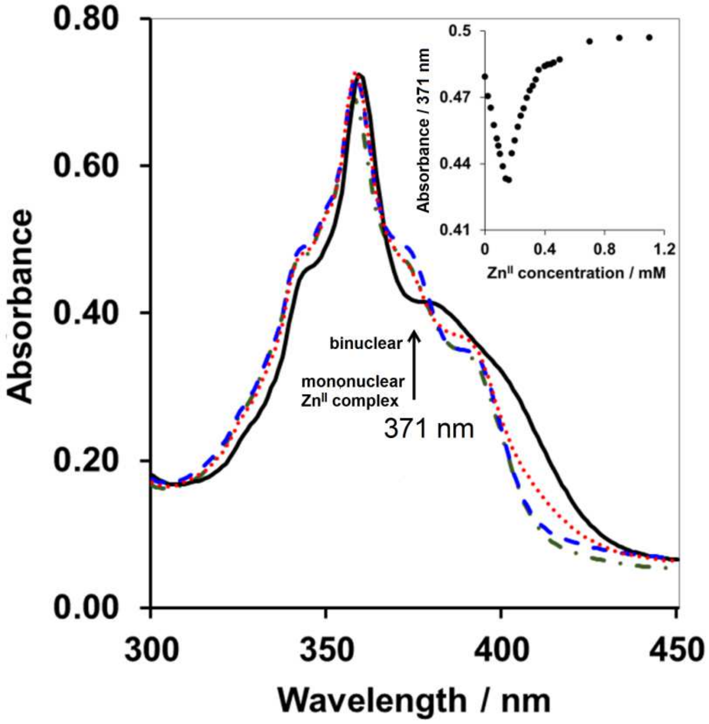

Figure S3. UV/Visible spectrum of ligand of **1** (0.16 mM) titrated with Zn^{II} at concentration of 0 (green dash dot line), 0.16 (black solid line), 0.32 (red round dot line) and 3.2 mM (blue dash line). Plot of UV absorption at 371 nm (inset) upon Zn^{II} titration in 10 mM HEPES buffer/MeOH = 1/1 (v/v).

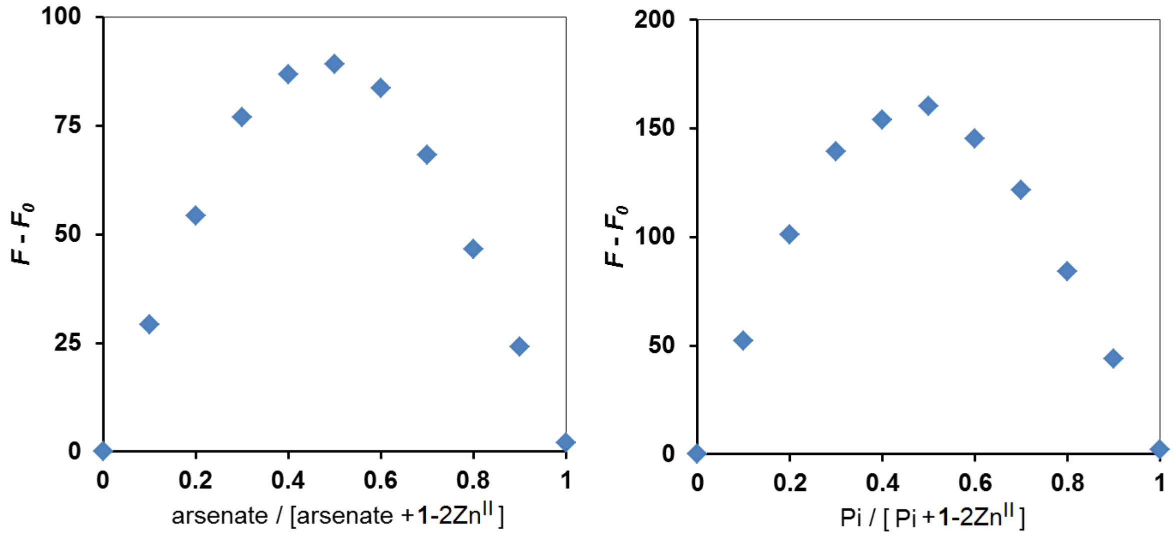

Figure S4. Job plots between **1** and arsenate (left) and phosphate (right). Measurement condition: 10 mM HEPES buffer (pH 7.2), 0.1 mM $ZnSO_4$, 25°C. λ_{ex} = 359 nm.

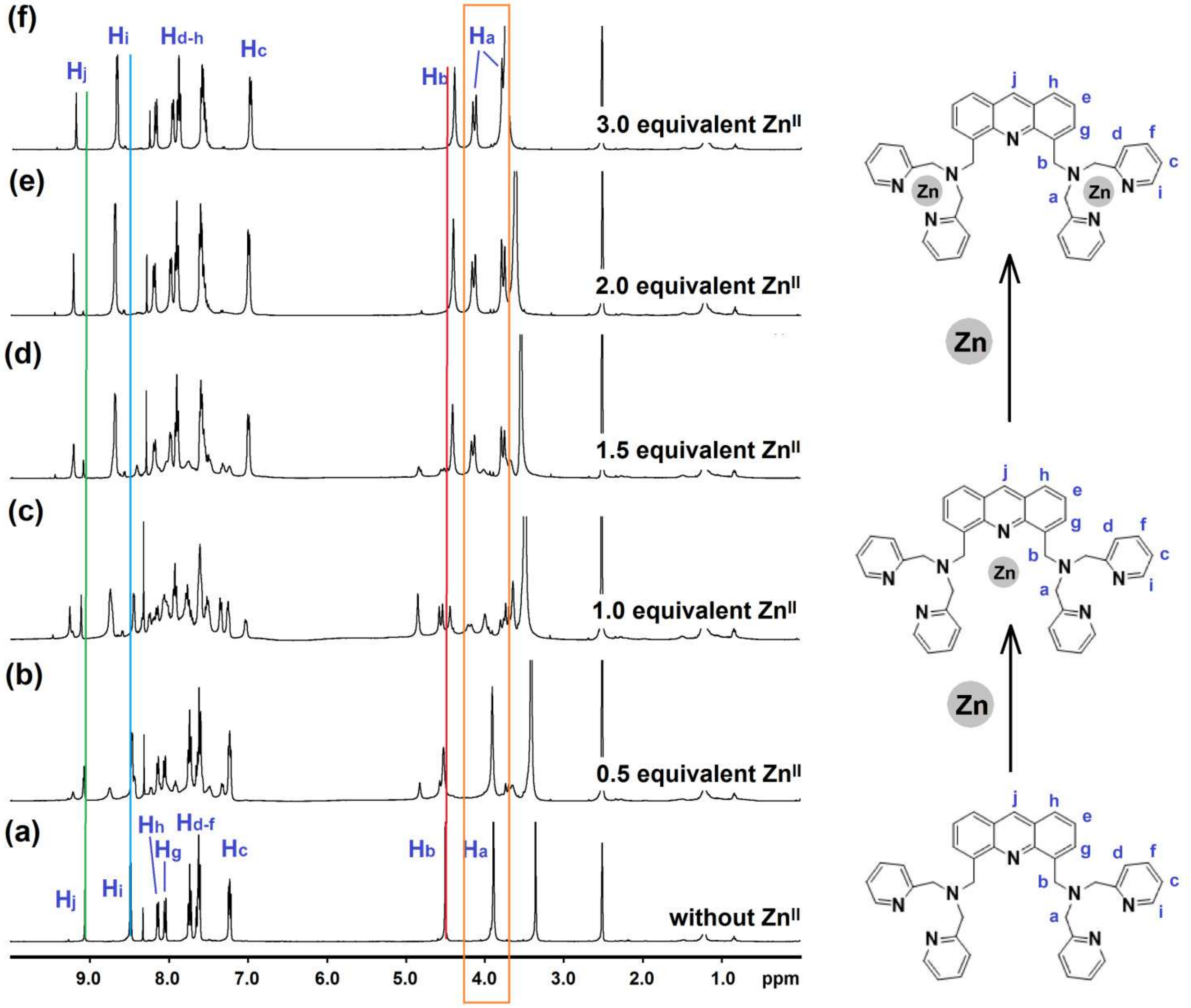

Figure S5. ^{1}H NMR (DMSO-d6) of the acridine Dpa ligand (30 mM) titrated with Zn^{II}

The ^{1}H NMR peak of Ha of the ligand (8H, s, δ 3.89) disappeared (Fig. S5a), and the new two Ha peaks of the Zn^{II} complex (4H, d, δ 3.75 and 4.14) were observed (Fig, S5f), indicating the coordination of Zn^{II} to the two Dpa moieties. The peak of Hb of the ligand (4H, s, δ 4.50) vanished in the mononuclear Zn^{II} complex (Fig. S5c), but appeared again as upfield shift (4H, s, δ 4.40) when the second Zn^{II} were fully coordinated to form binuclear Zn^{II} complex (Fig. S5e-f), suggesting that acridine nitrogen is involved in the coordination of Zn^{II} in the mononuclear Zn^{II} complex. The new downfield shift of Hi (4H, d, δ 8.48→8.67) and Hj (1H, s, δ 9.06→9.12) upon complexation of $2Zn^{II}$ was also observed.

It should be noted that under concentrated condition of the complex (30 mM) used in this ^{1}H NMR titration, the binuclear Zn^{II} complex was formed upon addition of 2 equivalent of Zn^{II}. However, under the extremely diluted condition of fluorescence measurement (5 μM, 6,000-fold diluted) the binuclear Zn^{II} complex was observed only when the excess Zn^{II} was added as clearly shown by fluorescence titration (Fig. S2) and UV-visible titration (Fig. S3).

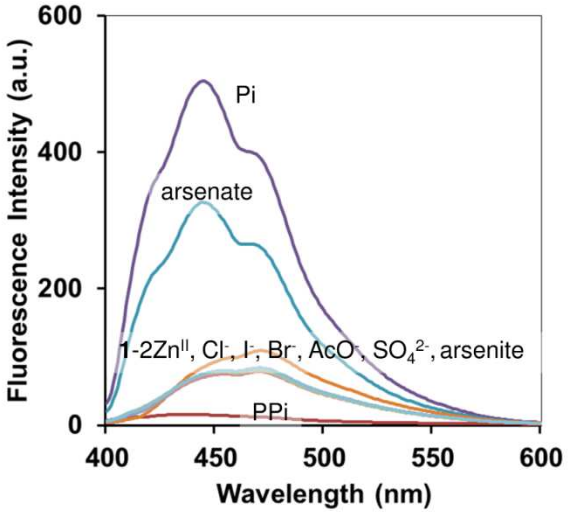

Figure S6. Fluorescence emission of **1** (5 μM) upon binding to arsenate, Pi and other potential interferences at 20 μM. Measurement condition: 10 mM HEPES buffer (pH 7.2), 0.1 mM $ZnSO_4$, 25°C. λ_{ex} 359 nm.

Staining with Toluidine Blue. Staining of the plant sample with toluidine blue O was adapted from Mitra and Loqué (*J. Vis. Exp.* **2014**, 87, e51381). *Wolffia* sample was placed on a glass slide, to which 2 drops of toluidine blue O (0.04 % (w/v) ethanol solution) were gently added. The specimen was incubated for 1 min before rinsing by adding 2 drops of water, which were then removed by blotting at the edge of liquid. An additional drop of water was added and the cover slip was laid on top of the sample. Then the stained sample was imaged under fluorescence light microscope (Olympus BX51) with camera set (Olympus PM10-SP).

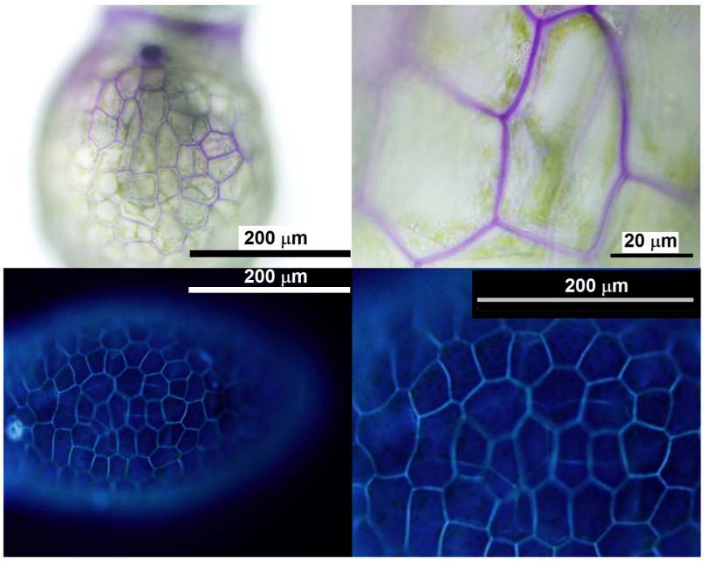

Figure S7. Staining of *Wolffia* with toluidine blue O (0.04 % (w/v) ethanol solution) (above) and **1** (1 mM) in the excess Zn^{II} for 30 min in dark at 27°C (below).
